# Transcriptional regulation of Sox2 by the retinoblastoma family of pocket proteins

**DOI:** 10.18632/oncotarget.2996

**Published:** 2014-12-18

**Authors:** Jéssica M. Vilas, Alba Ferreirós, Carmen Carneiro, Lluis Morey, Sabela Da Silva-Álvarez, Tânia Fernandes, María Abad, Luciano Di Croce, Tomás García-Caballero, Manuel Serrano, Carmen Rivas, Anxo Vidal, Manuel Collado

**Affiliations:** ^1^ Instituto de Investigación Sanitaria de Santiago de Compostela (IDIS), Complexo Hospitalario Universitario de Santiago de Compostela (CHUS), SERGAS, E15706 Santiago de Compostela, Spain; ^2^ Departamento de Fisioloxía and Centro de Investigación en Medicina Molecular (CIMUS), Universidade de Santiago de Compostela, Instituto de Investigaciones Sanitarias de Santiago de Compostela (IDIS), E15782 Santiago de Compostela, Spain; ^3^ Centre for Genomic Regulation and UPF, E08003 Barcelona, Spain; ^4^ Tumor Suppression Group, Spanish National Cancer Research Centre (CNIO), E28029 Madrid, Spain; ^5^ Institució Catalana de Recerca i Estudis Avançats (ICREA), E08010 Barcelona, Spain; ^6^ Departamento de Ciencias Morfológicas, Facultad de Medicina. USC. Complejo Hospitalario de Santiago (CHUS), SERGAS, E15706, Santiago de Compostela, Spain; ^7^ Departamento de Biología Molecular y Celular, Centro Nacional de Biotecnología-CSIC, E28049 Madrid, Spain; ^8^ Centro de Investigación en Medicina Molecular (CIMUS), Universidade de Santiago de Compostela, Instituto de Investigaciones Sanitarias de Santiago de Compostela (IDIS), E15706 Santiago de Compostela, Spain

**Keywords:** retinoblastoma, Sox2, stem cells, cancer

## Abstract

Cellular reprogramming to iPSCs has uncovered unsuspected links between tumor suppressors and pluripotency factors. Using this system, it was possible to identify tumor suppressor p27 as a repressor of *Sox2* during differentiation. This led to the demonstration that defects in the repression of *Sox2* can contribute to tumor development. The members of the retinoblastoma family of pocket proteins, pRb, p107 and p130, are negative regulators of the cell cycle with tumor suppressor activity and with roles in differentiation. In this work we studied the relative contribution of the retinoblastoma family members to the regulation of *Sox2* expression. We found that deletion of *Rb* or *p130* leads to impaired repression of *Sox2*, a deffect amplified by inactivation of *p53*. We also identified binding of pRb and p130 to an enhancer with crucial regulatory activity on *Sox2* expression. Using cellular reprogramming we tested the impact of the defective repression of *Sox2* and confirmed that *Rb* deficiency allows the generation of iPSCs in the absence of exogenous *Sox2*. Finally, partial depletion of Sox2 positive cells reduced the pituitary tumor development initiated by *Rb* loss *in vivo*. In summary, our results show that *Sox2* repression by pRb is a relevant mechanism of tumor suppression.

## INTRODUCTION

Cellular reprogramming to induced pluripotency by the combined action of defined transcription factors is a powerful *in vitro* system to uncover basic mechanisms governing stem cell biology. Given the similarity between cellular reprogramming and oncogenic transformation [1], others and us addressed the role of tumor suppressor genes opposing this process. In this manner, it was possible to identify crucial barriers impairing the efficient conversion of somatic cells to induced-pluripotent stem cells imposed by well-known tumor suppressor genes such as those encoded by the *p53* gene and the *Ink4a/Arf* locus [2-6].

Further studies on the effect of other tumor suppressor genes in the process of cellular reprogramming allowed us to identify an unprecedented connection between a cell cycle regulator, p27, and a pluripotency factor, Sox2 [7]. A previously unrecognized transcriptional regulatory activity of p27 allows the efficient repression of *Sox2* in differentiated cells. Loss of this regulation leads to unscheduled expression of *Sox2* in differentiated cells with the drastic phenotypic consequences that characterize *p27*-null mice: gigantism, pituitary tumor development, and retinal defects. The transcriptional regulation of *Sox2* during differentiation is exerted by a partially characterized repressive complex in which, apart from p27, we identified Sin3a, E2F4, and the pocket protein family member p130.

The retinoblastoma family of pocket proteins (composed by pRb, p107 and p130) is a family of negative regulators of the cell cycle with structural homology, and with overlapping and unique functions. The product of the *RB* gene is considered the member of the family with the most relevant tumor suppressor activity, since it is inactivated in a large number of human cancers [8-12]. In addition, its deletion in mice results in tumors, mainly of the pituitary and the thyroid gland [13]. The other two family members have also been involved in cancer development, although their relevance is secondary compared to pRb [13]. In addition to its role in cancer, pRb controls cellular differentiation during embryonic development and in adult lineages [14], with p107 and p130 also playing regulatory roles in adult cell differentiation [15].

Our previous results investigating the transcriptional repression of *Sox2* by p27 in which the pocket protein family member p130 was part of the repressive complex, together with the similarities between the process of reprogramming to pluripotency and oncogenic transformation, prompted us to investigate the role of the pocket protein family members in the transcriptional repression of *Sox2*. We found that deletion of *Rb* or *p130* leads to impaired repression of *Sox2*, while loss of *p107* has no apparent effect. Furthermore, we identified binding of pRb and p130 to an enhancer downstream of *Sox2* important for its regulation, the *Sox2-SRR2* enhancer, a region that showed alterations in epigenetic marks upon loss of pocket protein family members. The functional consequences of the defective repression of *Sox2* are manifested in the cellular reprogramming of *Rb* deficient cells to iPSCs in the absence of exogenous Sox2, and *in vivo* in *Rb* loss-driven pituitary tumor development.

## RESULTS AND DISCUSSION

### Cells lacking *Rb* or *p130* express higher levels of *Sox2*

**Figure 1:**
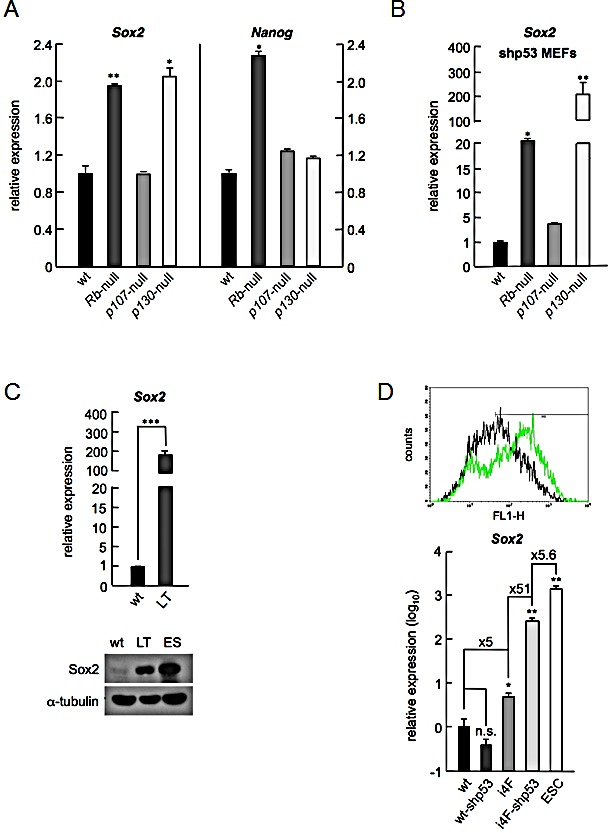
Cells lacking *Rb* or *p130* express higher levels of *Sox2* a, *Sox2* (left) or *Nanog* (right) mRNA levels in wt, *Rb*-null, *p107*-null, and *p130*-null primary MEFs as assessed by TaqMan expression analysis. Absolute values are referenced to the levels obtained with primary wt cells. b, *Sox2* mRNA levels in wt, *RB*-null, *p107*-null, and *p130*-null immortalized fibroblasts assessed as in (a). Absolute values are referenced to the levels obtained with wt cells. c, *Sox2* mRNA (upper panel) and protein expression by Western blot (lower panel) in cell extracts from wt and SV40 large-T antigen (LT) immortalized fibroblasts. Absolute values are referenced to the levels obtained with wt cells. d, Graph showing the analysis of GFP expression by flow cytometry from EOS pluripotency reporter plasmid introduced in reprogrammable primary (i4F-MEFs, black line) or immortalized (i4F-MEFs-shp53, green line) cells (upper panel). The settings were previously adjusted to consider i4F-MEFs without EOS plasmid as GFP negative. *Sox2* mRNA levels expressed by wt or immortalized wt-shp53 MEFs, i4F or immortalized i4F-shp53 MEFs, and ESCs, measured by qRT-PCR (lower panel). Values are referenced to the levels obtained using wt primary MEFs and in log_10_ scale. All data correspond to the average ± s.d. of qRT-PCR data. Statistical significance was assessed by the two-tailed Student's t-test: *** *p* < 0.001; ** *p* < 0.01; * *p* < 0.05; n.s. non significant.

We previously showed that cell cycle regulator p27 contributes to the transcriptional repression of *Sox2* together with pocket protein p130 and consequently, *p27* deficient cells or shRNA-mediated knockdown of *p130* leads to an increase of *Sox2* expression that has measurable phenotypic consequences [7]. To study in more detail the relative potential contribution of the pocket protein family members to the repression of pluripotency genes, and specifically to the regulation of *Sox2*, we measured by qRT-PCR the mRNA levels of *Sox2* and *Nanog* in early passage primary mouse embryo fibroblasts (MEFs) derived from *Rb*-, *p107*-, or *p130*-null mice. MEFs do not express detectable levels of these pluripotency genes or their levels of expression are negligible. In contrast, the absence of *Rb* or *p130* resulted in moderate but reproducibly increased levels of *Sox2* mRNA (Figure 1A, left panel). Surprisingly, cells lacking *Rb* also showed increased levels of *Nanog* (Figure 1A, right panel), a pluripotency gene normally not expressed in differentiated cells.

Strikingly, when we analyzed the expression of *Sox2* in MEFs from the same genotypes immortalized by an shRNA targeting *p53* (shp53) we observed an even higher increase in the expression of *Sox2* specifically for MEFs deficient in *Rb* or *p130*, but importantly, not in wt or *p107*-null cells (Figure 1B). In contrast, the increased expression of *Nanog* remained constant in *Rb*-deficient MEFs even after immortalization with shp53. Confirming these results, we checked *Sox2* levels in MEFs immortalized by the expression of viral oncoprotein large-T (LT) antigen from SV40. LT is known for its ability to block several cellular functions and prominently among them, the tumor suppressor pathways controlled by p53 and the three pocket proteins [16]. In agreement with the above results, MEFs immortalized by LT showed an increase in the expression of *Sox2* mRNA by qRT-PCR (Figure 1C, upper panel), with levels high enough to allow clear detection of Sox2 by Western blot (Figure 1C, lower panel). Knockdown of *p53* after shp53 expression is not causing an increase of *Sox2* in wt cells and this seems to be occurring only in cells deficient in *Rb* or *p130*, that already underwent some form of deregulation in the expression of *Sox2*. To further explore this, we used reprogrammable MEFs derived from a transgenic mouse carrying an inducible cassette for the expression of the four reprogramming factors, *Oct4*, *Sox2*, *Klf4* and *c-Myc* (i4F-MEFs) [17]. First we introduced a pluripotency reporter plasmid carrying an active promoter derived from a mouse early transposon (ETn) that is specific for undifferentiated pluripotent stem cells, combined with *Oct4*- and *Sox2*-binding motifs (EOS)[18]. Reprogrammable MEFs carrying this EOS reporter plasmid showed a low level expression of GFP, indicative of a minor leaky expression from the transgenic cassette. When we expressed the shRNA against *p53* in these i4F-MEFs carrying the EOS reporter plasmid a clear increase in the expression of GFP was evident by inspection under a fluorescent microscope. To verify and quantify this observation, we analyzed GFP expression in these cells by flow cytometry (Figure 1D, upper panel). In this manner, we could confirm the visual observation both regarding the alteration of the proportion of GFP positive cells (51.7% in i4F-MEFs versus 71.1% in i4F-MEFs-shp53), and in the intensity of GFP expression (mean fluorescence of 116.38 in i4F-MEFs versus 265.09 in i4F-MEFs-shp53). In agreement with the fluorescence data, these cells showed by qRT-PCR a low level of *Sox2* expression that was above the levels observed in wt cells. These low levels of *Sox2* expression were sharply increased when shp53 was introduced in i4F-MEFs but not in wt cells suggesting, again, that decreasing the levels of p53 amplifies the already deregulated expression of *Sox2* (Figure 1D, lower panel), even when this initial deregulation comes from transgenic expression. The increased levels observed after knockdown of *p53* were produced not from the transgene but from the endogenous *Sox2* locus, since they were detected using a specific pair of oligonucleotides for the endogenous *Sox2*. These results imply that a low level expression of *Sox2* would be sufficient to activate its own endogenous transcription provided that p53 is downregulated, and point to a novel function for p53 blocking upregulation of *Sox2* in differentiated cells.

Taken together, these results show a defective repression of *Sox2* transcription in the absence of *Rb* or *p130* that results in low but reproducible levels of this pluripotency gene in primary cells. Lack of *p53* leads to higher expression levels of *Sox2* only when they are originally deregulated by deficiency in *Rb* or *p130*.

### pRb and p130 bind to the *Sox2*-*SRR2* and their absence alters the epigenetic marks on the enhancer

**Figure 2:**
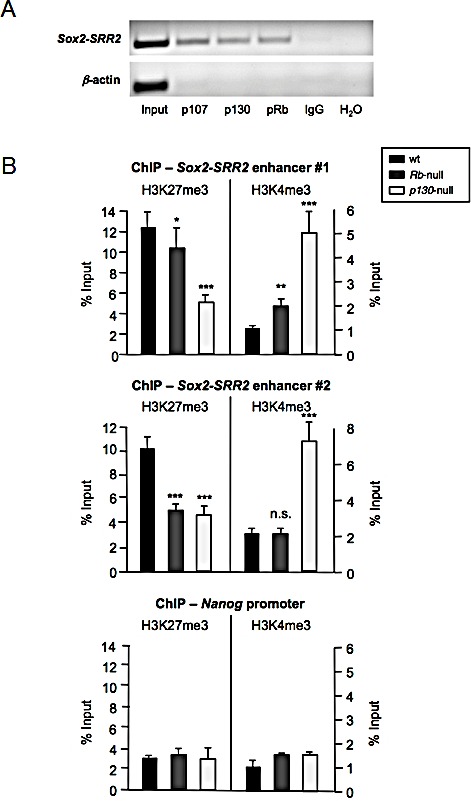
Binding of the Rb family of pocket proteins to the *Sox2*-*SRR2* enhancer and effect of their absence on histone marks a, Chromatin immunoprecipitation (ChIP) assay using antibodies against p107, p130, and pRb followed by semi-quantitative PCR using primers amplifying the *Sox2-SRR2*. Primers amplifying β-actin promoter and IgG were used as negative controls. b, ChIP of repressive H3K27me3 and active H3K4me3 histone marks in the *Sox2-SRR2* enhancer of wt, *Rb*-null, and *p130*-null MEFs, using two different sets of primers amplifying the *Sox2-SRR2* enhancer (upper and middle panels). Control ChIP assay using primers amplifying *Nanog* promoter is shown at the bottom panel. All data correspond to the average ± s.d. of qPCR data. Statistical significance was assessed by the two-tailed Student's t-test: *** *p* < 0.001; ** *p* < 0.01; * *p* < 0.05; n.s. non significant.

The main regulatory element of *Sox2* in pluripotent stem cells is an enhancer located downstream of the single coding exon of *Sox2* gene, called *SRR2*, bound by Sox2 itself and Oct4 to positively drive its expression during pluripotency [19]. Upon differentiation, Sox2-Oct4 on *SRR2* are displaced by a repressive complex formed by, at least, p27, Sin3a, E2F4, and the pocket protein family member p130 that permanently occupies this regulatory element in somatic cells [7]. Based on this, we decided to look for the binding of pRb, p107, and p130 in differentiated cells by chromatin immunoprecipitation (ChIP) using extracts from MEFs. Chromatin precipitated using antibodies against pRb, p107 or p130 produced a clearly distinguishable band when subjected to PCR using oligonucleotides amplifying *Sox2-SRR2* (Figure 2A). In contrast, control IgG did not produce an amplification band. This result implies that the three Rb family pocket proteins have the potential to bind to *Sox2-SRR2*. However, as shown above (Figure 1A), deficiency of p107 does not lead to increased expression of *Sox2*, probably as a result of compensatory mechanisms by the other members of the family. Functional compensation among pocket proteins is a common feature of the family [13].

Absence of p27 from the repressive complex binding to *Sox2-SRR2* leads to an alteration of the active and repressive histone marks present in the enhancer [7]. Given the identification of pRb and p130 bound to *Sox2-SRR2* and the derepression of *Sox2* observed in these cells, we wondered whether a similar situation might be also occurring in the absence of these pocket proteins. To address this issue we performed ChIP assays with antibodies against the repressive mark H3K27me3 and the active mark H3K4me3 in cell extracts from MEFs deficient for pRB or p130, followed by qRT-PCR to quantify the presence of *Sox2-SRR2* in the immunoprecipitates. Using two different sets of oligonucleotides amplifying *Sox2-SRR2* we obtained similar results (Figure 2B). The repressive H3K27me3 histone mark seems to be reduced in cells deficient for the *Rb* and *p130* pocket protein family members, while the active H3K4me3 mark appears increased in the absence of *p130* with the two sets of primers, and in the absence of *Rb* at least when using one of the two sets of primers. In contrast, ChIP for these same histone marks in the *Nanog* promoter did not show any alteration (Figure 2B). These changes support the notion that lack of *Rb* or *p130* has a specific impact on the epigenetic marks around the crucial *Sox2-SRR2* regulatory element of *Sox2*.

### Cells lacking *Rb* can be reprogrammed without ectopic Sox2

**Figure 3:**
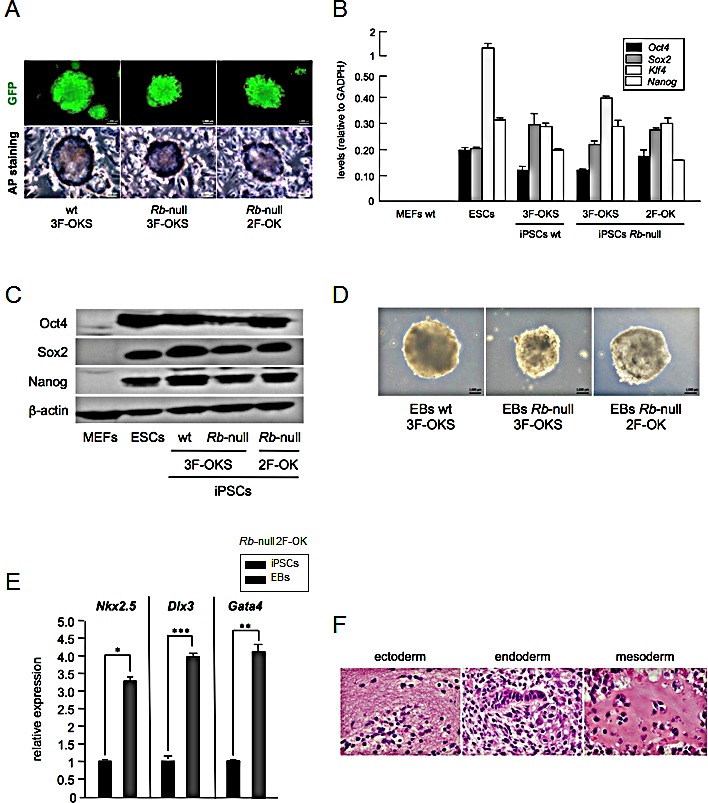
Absence of *Rb* allows two-factor (Oct4 and Klf4) reprogramming a, Representative pictures of iPSC colonies expressing GFP from the EOS pluripotency reporter plasmid (top panels), and stained for alkaline phosphatase (AP, bottom panels). Shown are iPSC colonies obtained in wt MEFs after three-factor expression (Oct4, Klf4, and Sox2; 3F-OKS; left panel), and *Rb*-null MEFs after three-factor (3F-OKS; middle panel) or two-factor (Oct4, Klf4; 2F-OK; right panel) expression. b, Pluripotency factor (*Oct4*, *Sox2*, *Klf4*, and *Nanog*) mRNA expression by qRT-PCR in iPSCs obtained from wt primary MEFs reprogrammed by 3F-OKS, or *Rb*-null with 3F-OKS or 2F-OK. Null expression from MEFs is shown as negative control, and expression in ESCs as positive control. c, Western blot analysis of the expression of pluripotency factors (Oct4, Sox2, and Nanog) in the same set of cells as in (b). d, Representative pictures of embryoid bodies (EBs) obtained after *in vitro* spontaneous differentiation of iPSCs generated from wt primary MEFs reprogrammed by 3F-OKS, or *Rb*-null with 3F-OKS or 2F-OK. e, Differentiation factor (*Nkx2.5*, *Dlx3*, and *Gata4*) mRNA expression by qRT-PCR in EBs obtained from iPSCs generated from *Rb*-null primary MEFs reprogrammed by 2F-OK. Values are referred to the expression obtained for the corresponding iPSCs. All data correspond to the average ± s.d. of qRT-PCR data. Statistical significance was assessed by the two-tailed Student's t-test: *** *p* < 0.001; ** *p* < 0.01; * *p* < 0.05. f, Representative pictures of H&E stained sections of EBs obtained from iPSCs generated from *Rb*-null primary MEFs reprogrammed by 2F-OK, and embedded in paraffin. Pictures show examples of ectodermal, endodermal and mesodermal differentiation.

Given the low level expression of *Sox2* present in primary MEFs deficient for *Rb* we wondered whether this deregulated expression could have a phenotypic consequence that could be exposed through the use of the cellular reprogramming system. For this, we performed a cellular reprogramming experiment on wt, *Rb*, *p107*, or *p130* deficient MEFs, with ectopic expression of Oct4, Klf4 and Sox2 (3F-OKS), or omitting Sox2 (2F-OK). Control reprogramming of wt MEFs with 3F-OKS worked as anticipated giving rise to alkaline-phosphatase-positive (AP+) iPSC colonies after 14 days with the typical morphology and at the expected rate. As predicted, wt cells failed to produce any colonies when only 2F-OK were used in the reprogramming experiments. In contrast, when we used *p130* deficient cells we obtained colonies both with 3F-OKS and with 2F-OK, although 2F-OK produced colonies at much lower frequency, as reported [7]. Cells lacking *p107* could be reprogrammed at approximately the same rate as wt cells with 3F-OKS while no colonies emerged when using 2F-OK despite repeated efforts. Interestingly, when we used MEFs *Rb*-null we obtained AP+ iPSC colonies not only with 3F-OKS, as expected, but also when using 2F-OK, the combination omitting *Sox2* (Figure 3A). The efficiency and kinetics of colony formation with 2F-OK were very low, similarly to what was previously reported and we confirmed when *p130* deficient cells were used. To further prove the pluripotency of the iPSC colonies obtained, we introduced the EOS pluripotency reporter plasmid. EOS lentiviral transduction of MEFs prior to the expression of the reprogramming factors allowed us to observe the emergence of GFP positive iPSC colonies from 2F-OK reprogrammed *Rb*-null MEFs (Figure 3A). These colonies were picked and expanded in stem cell culture conditions and the mRNA of *Oct4*, *Sox2*, *Klf4*, and *Nanog* was analyzed by qRT-PCR, confirming the expression of the pluripotency factors (Figure 3B). Similarly, Western blot analysis of protein extracts from these iPSC colonies showed the presence of Oct4, Sox2, and Nanog proteins (Figure 3C). Our *Rb*-null derived iPSCs with 2F-OK showed similar levels of mRNA or protein for all the pluripotency markers tested than iPSCs obtained from wt or *Rb*-null MEFs reprogrammed using 3F-OKS, or than control embryonic stem cells (ESCs).

Next we tested the capacity of the 2F-OK iPSCs from *Rb*-null cells to form embryoid bodies (EBs) in culture. For this, we used the standard hanging drop method to form cell aggregates that were further cultured in non-adherent conditions until they spontaneously showed contractile activity, indicative of cardiomyocyte differentiation. The number and size of the EBs obtained with 3F-OKS in wt or *Rb*-null, as well as with 2F-OK in *Rb*-null, was similar (Figure 3D). EBs were collected and RNA was extracted and analyzed by qRT-PCR for markers of differentiation along the three germ layers endoderm, mesoderm and ectoderm, confirming the pluripotent capacity of the iPSCs obtained (Figure 3E). In addition, we analyzed histologically the structures generated within the EBs and observed features of differentiation to the three germ layers (Figure 3F).

These results show that the deregulated expression of *Sox2* in *Rb* deficient cells is sufficient to allow cell reprogramming to iPSCs without the need for exogenous *Sox2*, demonstrating that this increased expression has relevant functional consequences.

### Partial depletion of Sox2 positive cells alleviates the pituitary tumor phenotype caused by reduced *Rb* in mice

**Figure 4:**
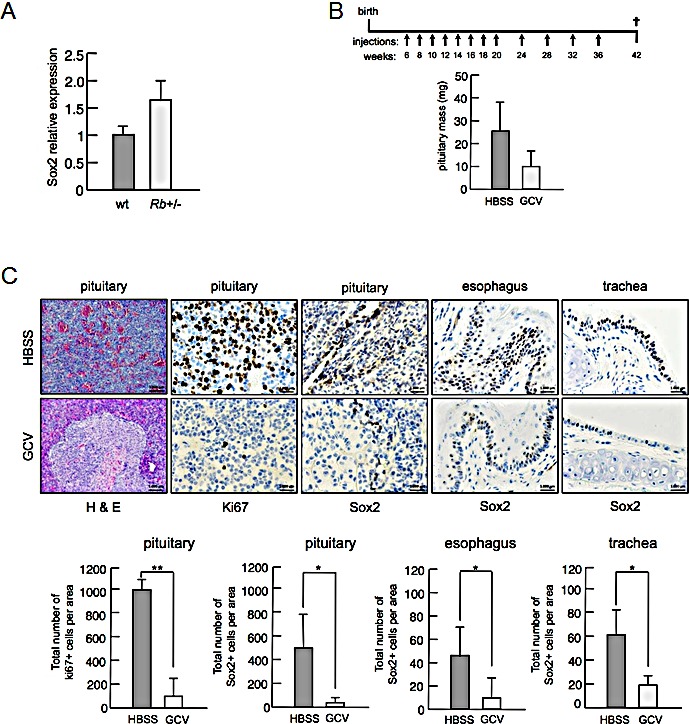
*In vivo* effect of partial depletion of Sox2 positive cells in *Rb+/−* mice a, *Sox2* mRNA levels in the pituitary of wt (n=4) and *Rb*-het (n=4) mice. b, Schematic representation of the experimental protocol of gancyclovir (GCV) treatment of compound *Rb*-het/Sox2-TK mice (upper panel). Intraperitoneal injections were administered at the times indicated (weeks) and mice were sacrificed when they were 42 weeks old. Pituitary mass (n=4 for HBSS; n=3 for GCV) of *Rb*-het/Sox2-TK mice (lower panel). Means ± s.e.m. are shown. c, Representative pictures of paraffin sections from control HBSS-treated (upper panels) or GCV-treated (lower panels) tissues from *Rb*-het/Sox2-TK mice. The first three rows of pictures correspond to pituitary sections stained for H&E, proliferative marker Ki67 and Sox2, respectively. The two rows of pictures at the most right part correspond to control tissues, esophagus and trachea, stained for Sox2. Below is shown the quantification of Ki67 and Sox2 positive cells.

Human *SOX2* gene locus at 3q26.3 is amplified in several cancer types including glioblastoma, small-cell lung cancer (SCLC) and many forms of squamous cell carcinoma (SCC) [20]. However, the involvement of Sox2 in cancer is not restricted to tumors showing gene amplification, opening up the possibility of other regulatory mechanisms contributing to the potential role of Sox2 in cancer. In this sense, we previously reported the contribution of Sox2 deregulated expression to the development of pituitary tumors in mice deficient for *p27* [7]. Our current results were reminiscent of this same situation. Therefore, we wanted to determine if the incomplete repression of *Sox2* expression in differentiated cells lacking *Rb* could contribute to tumor development. Heterozygosity of *Rb* in the mouse leads to the development of pituitary tumors after stochastic loss of the second *Rb* allele [21]. Given our previous results, we decided to measure *Sox2* mRNA expression in pituitary tissue from wt or *Rb*-het mice and observed a modest increase in the expression of *Sox2* (Figure 4A) consistent with our previous findings in *Rb*-null MEFs (Figure 1A).

To test if Sox2 positive cells contribute to the emergence of these tumors, we took advantage of a previously reported mouse model in which the thymidine kinase gene (TK) has been inserted into the endogenous *Sox2* locus, the Sox2-TK mice [22]. Exposure of Sox2 positive cells to the drug gancyclovir (GCV) induces cell death. Persistent exposure to GCV results in animal death after 1-2 weeks. However, we observed that single intraperitoneal injection of GCV every 2 weeks is well tolerated by these mice and results in various degrees of Sox2 positive cell depletion. Based on this, we generated compound *Rb*-het/Sox2-TK mice and subjected them to a repetitive GCV administration protocol in order to test the contribution of Sox2 positive cells to tumors initiated by *Rb* deficiency (Figure 4B). When the treatments were completed we sacrificed the animals and extracted tissues for histological analysis. Pituitary masses were measured and we observed a tendency to a reduced size of pituitaries from *Rb*-het/Sox2-TK animals in the GCV-treated group compared to the HBSS-treated control group (Figure 4B). Then, we verified that we produced different degrees of Sox2 positive cell depletion by the GCV administration, as judged by Sox2 immunohistochemical staining in sections from control tissues, esophagus and trachea. Despite the low number of animals analyzed, we observed that when the depletion was efficient, *Rb*-null pituitaries showed a nearly normal morphology with reduced number of Sox2 positive cells and low cell proliferation, as judged by Ki67 staining (Figure 4C).

Although further studies would be required, these results are in line with our previous observations for reduced pituitary size and tumor development in *p27*-null mice in the context of lower *Sox2* expression using *Sox2* heterozygous mice [7]. Interestingly, although Rb is ubiquitously expressed and its inactivation is an extremely frequent event in cancer, *Rb* deletion in mice is a tumor-initiating event only for some specific cell types. In this sense, it is tempting to speculate that *Rb* loss might result on pro-tumorigenic expression of *Sox2* only for these cell types. Even more, according to our results concomitant inactivation of *p53* might have synergistic effects on cancer by enhancing deregulated expression of genes such as *Sox2* whose upregulation might be initiated by the loss of *Rb*.

Taken together, our results unveil an unprecedented mechanism of tumor initiation mediated by loss of the proper strict control exerted by the retinoblastoma family of pocket proteins on the repression of genes, such as *Sox2*, with crucial functions in regulating the properties of adult stem/progenitor cells.

## MATERIALS AND METHODS

### Mice and cells

*Rb*+/− [21], *p107*−/− [23], *p130*−/− [24], Sox2-TK [22], and i4F mice [17] have been previously described. All genotypes were maintained on a C57BL/6 and 129SvJ hybrid background and all comparisons were made among mice derived from the same sets of crosses, and they therefore shared the same genetic background. Animals were kept under SPF conditions and all experiments were carried out under approval of the Santiago de Compostela University Bioethics (protocol number 15005AE/07/01/02/05C/AVF2) in compliance with Principles of Laboratory Animal Care of national laws.

Primary mouse embryo fibroblasts (MEFs, passage 1-2) were obtained from embryos of the indicated genotypes, as described previously [25]. Immortalization was achieved by expression of an shRNA targeting mouse p53 [26]. Both primary and immortalized fibroblasts were cultured in standard DMEM medium (Sigma) supplemented with 10% FBS (Sigma).

ES cells and iPS cells were cultured on top of feeder layers in DMEM (Sigma) supplemented with serum replacement (KSR, 15%, LifeTechnologies), LIF 1000 u/ml (Millipore), 1x Non-Essential Amino Acids (LifeTechnologies), 1% L-glutamine (Sigma), 1% Streptomycin/Penicillin (Sigma), and 0.1 mM 2-mercaptoethanol (LifeTechnologies).

### Protein and RNA expression analysis

For protein expression analysis, cell extracts were prepared using RIPA buffer (150mM NaCl, 10mM Tris-HCl pH 7.5, 0.1% SDS, 1% Triton X100, 5 mM EDTA pH 8.0, 1% Deoxycholate and sodium salt containing protease inhibitors), and appropriate volumes of cell extracts, adjusted to represent the same amount of total cellular protein (40 μg), were electrophoresed in 12% polyacrylamide gels. After electrophoretic transfer to a PVDF membrane at 100 V for 1 h at 4°C, the membrane was blocked with 5% milk in TTBS (20mM Tris-HCl pH7.5, 150mM NaCl, 0.05% Tween-20) for 1 h at room temperature. Membranes were incubated at 4ºC overnight with primary antibodies against SOX2 (SantaCruz, sc-17320; 1:500), OCT4 (SantaCruz, sc-9081; 1:500), or NANOG (Millipore, AB 5731; 1:5000). Incubation with the appropriate secondary antibodies conjugated to HRP was followed by visualization using the ECL system.

To measure RNA expression, total RNA was extracted using the NucleoSpin® RNA kit (Macherey-Nagel) following the indications of the provider and DNAse treatment. After nanodrop RNA quantification, the RNA was retrotranscribed into cDNA according to the manufacturer's protocol (High-Capacity cDNA Reverse Transcription Kit, Applied Biosystems). Quantitative Real Time-PCR was performed using SYBR Green Power PCR Master Mix (Applied Biosystems) in an Mx3005P real-time PCR system (Agilent technologies Stratagene). Relative quantitative RNA was normalized using the housekeeping gene *GADPH.* Primer sequences are available from the authors upon request. For specific experiments, TaqMan Real Time-PCR was performed using GoTaq® Probe qPCR Master Mix (Promega) in a StepOne™ and StepOnePlus™ PCR Real-Time System (Applied Biosystems). Relative quantitative RNA was normalized using the housekeeping gene *UBC*. The TaqMan® probes used were: Sox2 (Mm03053810_s1), Nanog (Mm02384862_g1).

### Chromatin immunoprecipitation

To identify the binding of the pocket proteins to the *Sox2-SRR2* enhancer, cells were harvested at 90% confluence and fixed by adding 1/10 of fixation solution (0,1M NaCl, 1mM EDTA, 0.5mM EGTA, 50mM Hepes pH8.0 and 11% Formaldehyde) to the culture media for 15 min at room temperature. Crosslinking was stopped by the addition of glycine to a final concentration of 0.125 M. Fixed cells were sonicated in lysis buffer (0.5% NP-40, 1% Triton X-100, 5 mM EDTA, 50 mM Tris-HCl pH 7.5, 150 mM NaCl plus protease inhibitors). For immunoprecipitation, 150 μg of chromatin were diluted in dilution buffer (1% Triton X-100, 0.1% sodium deoxycholate, 140 mM NaCl, 1 mM EDTA, 0.5 mM EGTA and 10 mM Tris-HCl, pH 8.0, containing protease inhibitors) and pre-cleared with blocked protein G-sepharose (GE Healthcare). An aliquot of 50 μg of chromatin was reserved as input. The antibodies used for the immunoprecipitation were pRb1 (Santa Cruz, C-15), p107 (Santa Cruz, C-18), p130 (Santa Cruz, C-20), and normal rabbit IgG (Santa Cruz, sc-2027). Immune complexes were precipitated with protein G-sepharose and washed sequentially with low-salt wash buffer (0.1% SDS, 1% Triton X-100, 2 mM EDTA, 20 mM Tris-HCl pH 8.1, 150 mM NaCl), high-salt wash buffer (0.1% SDS, 1% Triton X-100, 2 mM EDTA, 20 mM Tris-HCl pH 8.1, 500 mM NaCl), LiCl wash buffer (0.25 M LiCl, 1% NP-40, 1% deoxycholate-Na, 1 mM EDTA, 10 mM Tris-HCl pH 8.1) and TE buffer. Immunoprecipitated DNA was eluted in elution buffer (1% SDS, 0.1 M NaHCO3) and extracted using the Wizard SV Gel and PCR System (Promega). PCR was carried out using 5 μL of each sample except for the input that was previously diluted.

The primers used for PCR were:

*Sox2-SRR2*-F: 5′-ATTTATTCAGTTCCCAGTCCAAGC-3′

*Sox2-SRR2*-R: 5′-CCCTCTCCCCCCACGC-3′

β*-actin*-F: 5′-CAGTTCGCCATGGATGACGATATC-3′

β*-actin*-R: 5′-CCGCGAACCCGGCTTTGCACATG-3′

The analysis of histone marks was performed essentially as described [27]. Briefly, wt, RB-*null*, and p130-*null* MEFs were trypsinized and cross-linked in 1% formaldehyde (Sigma) for 10 min at room temperature (RT). Crosslinking was quenched with 0.125 M glycine for 5 min. Pelleted cells were lysed in 1 ml ChIP buffer and sonicated for 10 min in a Bioruptor (Diagenode). Soluble material was quantified by Bradford assays. 100 μg of chromatin were used to immunoprecipitate histone modifications. The antibodies H3K27me3 (Millipore, 07-449) and H3K4me3 (Diagenode, pAb003-050) were incubated overnight with the chromatin. Immunocomplexes were recovered with 30 μl of protein A or G-agarose bead slurry. Immunoprecipitated material was washed three times with low salt buffer and one time with high salt buffer DNA complexes were decrosslinked at 65°C for 3 h, and DNA was then eluted in 200 μl of water using the PCR purification kit (QIAGEN). Two microliters of DNA was used for each qPCR reaction using SYBR green (Fermentas).

The primers used for *Sox2-SRR2* were the ones shown above plus an extra set of primers:

*Sox2-SRR2*-F: 5′-CGTGGTAATGAGCACAGTCG-3′

*Sox2-SRR2*-R: 5′-AGGCTGAGTCGGGTCAATTA-3′

The primers used for *Nanog* promoter were:

*Nanog*-*promoter*-F: 5′-CAACTTACTAAGGTAGCCCGAGTCTTAA-3′

*Nanog*-*promoter*-R: 5′-CCTCCAAAAGTGCGGCTTT-3′

### Cellular reprogramming to iPSCs

Reprogramming of primary (passage 1-2) MEFs was performed using plasmids pMXs-Oct4, pMXs-Klf4, and pMXs-Sox2 [28] essentially as described [7]. Briefly, retroviral supernatants were produced in HEK-293T cells (3×10^6^ cells per 100-mm-diameter dish) transfected by the calcium chloride protocol with the ecotropic packaging plasmid pCL-Eco (5 μg) together with the reprogramming factors (5 μg) in combinations of 3 (Oct4, Klf4, and Sox2) or 2 (Oct4 and Klf4) factors. Retroviral supernatants (10 ml) were collected serially 48 h later and during 36 h, each time adding fresh medium to the cells (10 ml). The recipient MEFs had been seeded the previous day (1.4×10^6^ cells per 100-mm-diameter dish) and received 2.5 mL of each of the corresponding retroviral supernatants. This procedure was repeated every 12 hours for a total of 3 additions. After infection was completed, media was replaced by iPS cell medium (see above).

For alkaline phosphatase (AP) staining the iPSCs colonies were fixed with 4 % paraformaldehyde and the alkaline phosphatase activity was detected with BCIP (5-bromo-4-chloro-3-indolyl-phosphate)/NBT(nitro blue tetrazolium) Color Development Substrate (Promega) according to the manufacturer's instructions.

The lentiviral pluripotency reporter plasmid PL-SIN-EOS-C(3+)-EiP (Addgene #21313) [18] was used to visualize the emergence of iPSC colonies by inspection of GFP expression using a fluorescence microscope. Lentiviral transduction with this plasmid was performed after co-transfection of packaging vectors psPAX2 and VSVG in HEK-293T cells as described for retroviral infections.

### *In vitro* embryoid body differentiation

To test pluripotency of the iPSC colonies generated we performed *in vitro* embryoid body differentiation assays using the hanging drop technique. For this, iPSCs cells were trypsinized and counted. After removing trypsin, iPSCs were resuspended at a density of 2.5×10^5^ cells/mL in Embryoid Body medium: DMEM supplemented with 10% FBS, 1% Streptomycin/Penicillin, 1% L-Glutamine, 0.1 mM 2-mercaptoethanol and 1X Non-Essential Amino Acids. Small volumes of 20 μl were plated as droplets on the lid of the Petri dish and an average of 50 hanging drops were cultured over a cell culture dish containing PBS. After 3 days, droplets were collected and transferred to a Petri dish and further cultured in Embryoid Body medium for 15 days before harvested for qRT-PCR and histological analysis.

To analyze histological differentiation, EBs were included in melted agar and the resulting plug was fixed in buffered formalin at 4ºC, embedded in paraffin wax, and sectioned at a thickness of 5 μm. Sections were stained with hematoxylin and eosin for pathological examination.

### *In vivo* experiments

Starting at 6 weeks of age, compound *Rb*-het/Sox2-TK mice were intraperitoneally injected with gancyclovir (GCV) at 100 mg/Kg in HBSS (Cymevene, Roche Pharmaceuticals) or vehicle (HBSS) every 2 weeks during the first 20 weeks of life, and then switched to single injections every 4 weeks, to complete a total of 12 injections. Animals were sacrificed when they were 42 weeks old, tissues were removed, and pituitaries were weighted before processing for immunohistochemistry.

For immunohistochemical analysis, tissue was fixed in formalin at 4ºC, embedded in paraffin wax, and sectioned at a thickness of 5 μm. Sections were stained with hematoxylin and eosin for pathological examination or processed for immunohistochemical analysis with antibodies against mouse Ki67 (Master Diagnostica, SP6), or Sox2 (CST #3728, C70B1).
